# Compression Fracture of CFRP Laminates Containing Stress Intensifications

**DOI:** 10.3390/ma10091039

**Published:** 2017-09-05

**Authors:** Christian Leopold, Martin Schütt, Wilfried V. Liebig, Timo Philipkowski, Jonas Kürten, Karl Schulte, Bodo Fiedler

**Affiliations:** 1 Institute of Polymer and Composites, Hamburg University of Technology (TUHH), Denickestrasse 15, D-21073 Hamburg, Germany; timo.philipkowski@tuhh.de (T.P.); jonas.kuerten@googlemail.com (J.K.); schulte@tuhh.de (K.S.); fiedler@tuhh.de (B.F.); 2 CompriseTec GmbH, Steinhöft 5, D-20459 Hamburg, Germany; schuett@comprisetec.de; 3 Institute of Vehicle System Technology, Karlsruhe Institute of Technology (KIT), Rintheimer Querallee 2, D-76131 Karlsruhe, Germany; wilfried.liebig@kit.edu

**Keywords:** stacking sequence, scaling, free-edge, open-hole, compression after impact (CAI), fractography, scanning electron microscopy (SEM), kink-band

## Abstract

For brittle fracture behaviour of carbon fibre reinforced plastics (CFRP) under compression, several approaches exist, which describe different mechanisms during failure, especially at stress intensifications. The failure process is not only initiated by the buckling fibres, but a shear driven fibre compressive failure beneficiaries or initiates the formation of fibres into a kink-band. Starting from this kink-band further damage can be detected, which leads to the final failure. The subject of this work is an experimental investigation on the influence of ply thickness and stacking sequence in quasi-isotropic CFRP laminates containing stress intensifications under compression loading. Different effects that influence the compression failure and the role the stacking sequence has on damage development and the resulting compressive strength are identified and discussed. The influence of stress intensifications is investigated in detail at a hole in open hole compression (OHC) tests. A proposed interrupted test approach allows identifying the mechanisms of damage initiation and propagation from the free edge of the hole by causing a distinct damage state and examine it at a precise instant of time during fracture process. Compression after impact (CAI) tests are executed in order to compare the OHC results to a different type of stress intensifications. Unnotched compression tests are carried out for comparison as a reference. With this approach, a more detailed description of the failure mechanisms during the sudden compression failure of CFRP is achieved. By microscopic examination of single plies from various specimens, the different effects that influence the compression failure are identified. First damage of fibres occurs always in 0°-ply. Fibre shear failure leads to local microbuckling and the formation and growth of a kink-band as final failure mechanisms. The formation of a kink-band and finally steady state kinking is shifted to higher compressive strains with decreasing ply thickness. Final failure mode in laminates with stress intensification depends on ply thickness. In thick or inner plies, damage initiates as shear failure and fibre buckling into the drilled hole. The kink-band orientation angle is changing with increasing strain. In outer or thin plies shear failure of single fibres is observed as first damage and the kink-band orientation angle is constant until final failure. Decreasing ply thickness increases the unnotched compressive strength. When stress intensifications are present, the position of the 0°-layer is critical for stability under compression and is thus more important than the ply thickness. Central 0°-layers show best results for OHC and CAI strength due to higher bending stiffness and better supporting effect of the adjacent layers.

## 1. Introduction

In many applications fibre reinforced plastics (FRP) and especially carbon fibre reinforced plastics (CFRP) are increasingly used for weight optimization due to their density specific mechanical properties. However, their sudden and brittle failure behaviour and the complex damage mechanisms involved require often conservative design. A forecast when and where damage is initiated is thus very difficult, leading to high safety margins and limiting their potential for lightweight design. The compressive strength is often a design limit of CFRP, as it is significantly lower than the tensile strength. For the brittle fracture behaviour of CFRP under compression, no conclusive and entire theory exists, which describes all relevant mechanisms during failure, especially at stress intensifications.

**Figure 1:**
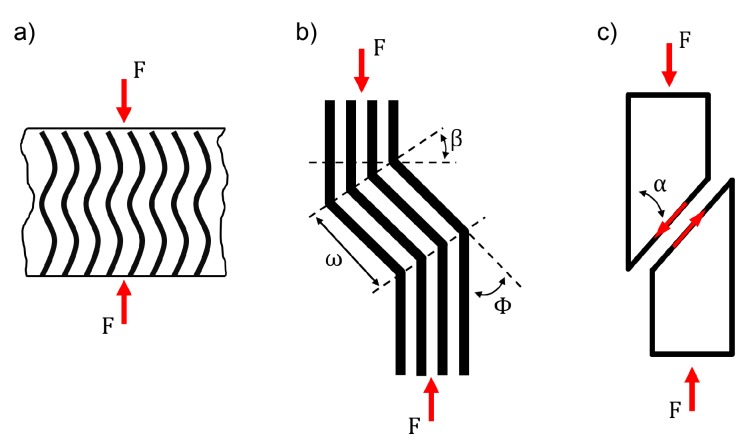
Scheme showing the different mechanisms contributing to compressive failure of FRP: (**a**) in-phase microbuckling; (**b**) kink-band geometry; (**c**) shear failure.

The first model for predicting compressive strength of composite laminates was presented by Rosen [1]. He proposed that compressive failure initiates due to fibre microbuckling and distinguished between two modes of microbuckling: in-phase microbuckling (shear mode) for higher and out-of-phase microbuckling (extension mode) for lower fibre volume fractions [1]. The in-phase microbuckling leads to the formation of a kink-band with increasing load. This is similar to the compressive failure of other fibrous materials like i.e., wood. Moran et al. [2] and Poulsen et al. [3] investigated the kinking failure in spruce and compared the damage process with that of CFRP. They identified three stages of compressive kinking. Incipient kinking as the first stage begins on a very small scale and is characterised by localised plastic shearing and buckling of fibres. In the following transient kinking stage the localised incipient kinking areas grow and coalesce to form a single dominant kink-band across the specimen. The last stage is steady state kinking during which the kink-band broadens laterally [2,3]. In CFRP these three stages occur as well in a similar process [2,4], but they are difficult to clearly identify because of the brittle fracture within a very short amount of time. Incipient kinking occurs when the matrix shear stress in fibre direction reaches a critical value [2]. The analytical model of Budiansky et al. [5,6] describes the initiation and propagation of such a kink-band with the orientation angle β, the kink-band width ω and the inclination angle Φ of the fibres. Gutkin et al. [7,8] described the initiation of microbuckling and the following kink-band as shear driven fibre failure with a distinct shearing angle α. Figure 1 sums up the different failure mechanisms of FRP under compressive loading with the nomenclature used in this work.

Initiation of kink-bands is facilitated at defects, e.g., voids [9,10] or local fibre misalignment [11]. Despite these flaws, the laminate properties and stacking sequence play an important role in damage initiation and propagation and the resulting mechanical properties under compressive loading. For composite laminates in general, size effects with regard to scaling of the specimens on the one hand and thickness of the constitutive plies on the other hand should be considered [12]. Soutis [13] and Lee and Soutis [14,15] investigated the influence of specimen and layer thickness on the strength and failure behaviour for unnotched compression (UNC) [13,14] and open-hole compression (OHC) [15,16] load cases and compared the compression behaviour of open-hole specimens with that under tensile loading and found a strength increase with decreasing dimensions or ply thickness [17]. Arteiro et al. [18] showed with micromechanical modelling and finite element simulation that the in situ effect in tension [19] exists as well under compression loading : They reported a higher ply strength with decreasing ply thickness and with increasing the stiffness of the surrounding layers [18]. These ply thickness effects become more and more important with the possibilities of the thin-ply technology by using plies below 50 μm of thickness. The damage mechanisms for standard and thin-ply laminates with regard to ply thickness and stacking sequence effects under compression are not yet fully clear [20,21] and require further research.

For composite laminates in many applications the influence of stress intensifications such as holes, notches or barely visible impact damage is critical. At holes or the edges of a laminate the free edge effect must be considered [22,23]. Due to high peel stresses, delamination between two layers initiates at the edges (resulting from a mathematical stress singularity [24]). With decreasing ply thickness, a suppression of these edge delaminations is reported [25]. Under compression fibre microbuckling initiates at the hole boundary followed by delamination and formation of a kink-band [26]. At free edges, compression failure is hence partly a result of delamination growth. With increasing hole diameter/specimen width ratio, compressive strength decreases [14,15,16,27]. The OHC failure process depend on interlaminar toughness. A high interlaminar toughness leads to a short crack rest after being initiated at the hole before brittle failure occurs, whereas a weaker interlaminar interface results in sudden failure [28]. Wang et al. [29] compared experimental results for open-hole tension and compression to predictions of a numerical finite element method (FEM) analysis and pointed out, that the compressive strength of a lay-up with subsurface 0°-plies is higher than that of a unidirectional (UD) lay-up due to higher stability of the load bearing fibres [29]. Thus, stacking sequence and optimum support of the 0°-layers carrying the highest load share is critical.

An open or filled hole is usually a design feature and can thus be considered for when designing a composite part. Impact events may occur during lifetime of a composite part and result in a stress intensification that is difficult to account for in the design process. Impact damage may be barely visible at the surface of a FRP laminate but result in severe damage such as matrix cracking, fibre breakage and delamination. Hence, the introduction of impact damage and compression after impact (CAI) properties are design limits. In addition, stacking sequence and scaling of the constitutive layers have an influence on resistance against impact damage [30,31,32,33]. A comparison between ply-block scaled and sublaminate scaled laminates reveals that the increase of interfaces available for delamination in the distributed plies of sublaminate scaled laminates results in more, but less large delaminations. If the number of interfaces available for delamination is reduced, larger delaminations occur [25]. This may be beneficial for thin-ply laminates with distributed plies, because of the high number of interfaces. Impact tests with different types of thin-ply laminates exhibit equal [20] or larger [21,30] delamination areas after impact with decreasing ply thickness. CAI strength is slightly improved with a significant decrease in ply thickness [30,34], with the delamination being less severe [20].

Delamination size is critical under compression as the constitutive layers are not supported at the delaminated areas. Fractography investigations from Greenhalgh et al. [35] about delamination growth and migration revealed that migration between different interfaces is important, as it is the slower propagating mechanisms, resulting in a smaller projected damage area. When delamination driving force direction and fibre orientation of the adjacent layer are in the same axis this results in fast delamination growth with larger damage areas [35]. In compression, delamination growth is preferably at interfaces with plies transverse to the loading direction, thus the 90°-layers are most critical for delamination propagation.

The subject of this work is an experimental investigation on the influence of ply thickness and stacking sequence in quasi-isotropic (QI) CFRP laminates containing stress intensifications under compression loading. The aim is to identify and discuss the different effects that influence the compression failure and the role the stacking sequence has on damage development and the resulting compressive strength. The influence of stress intensifications is investigated in detail at a hole in OHC tests, because with this type of stress intensification the exact shape as well as the stress state is well known and an interrupted test approach allows to identify the mechanisms of damage initiation and propagation from the free edge of the hole. CAI tests are executed in order to compare the OHC results to a different type of stress intensifications. UNC tests are carried out for comparison as a reference. We present an approach to use open-hole specimens for causing a distinct damage state and examine it at a precise instant of time during fracture process. Focus is thus set on failure initiation and propagation as well as the resulting final failure mode in OHC. With this method, a more detailed description of the failure mechanisms during the brittle compression failure of CFRP is achieved. In addition, the influence of the 0°-layer position with regard to damage initiation and the resulting mechanical properties is examined. The results contribute to a better understanding of the complex failure mechanisms under compression loading and may help to design composite parts accordingly.

## 2. Materials and Methods

### 2.1. Materials and Sample Preparation

The UD prepreg HexPly-M21/34%/UD194/T800S (Hexcel, Stade, Germany) is used for producing specimens for UNC, OHC and CAI tests with a quasi-isotropic lay-up of ${\left[45{{}^{\circ}}_{n}/0{{}^{\circ}}_{n}/-45{{}^{\circ}}_{n}/90{{}^{\circ}}_{n}\right]}_{\;ms}$ and ${\left[45{{}^{\circ}}_{n}/90{{}^{\circ}}_{n}/-45{{}^{\circ}}_{n}/0{{}^{\circ}}_{n}\right]}_{\;ms}$, with n=1,3 and m=3,1 respectively, in order to investigate the influence of both, the layer thickness and the position of the 0°-layers (stacking sequence). Thickness of the laminates is 4.56 mm with a theoretical cured ply thickness (CPT) of 190 μm. In addition, laminates from M21-34-12K-T800S-134 prepreg (Hexcel, Germany) and from 977-2-34-24A-IMS-268 prepreg (Hexcel, Germany) with a quasi-isotropic lay-up of ${\left[45{}^{\circ}/0{}^{\circ}/-45{}^{\circ}/90{}^{\circ}\right]}_{\;4s}$ respectively ${\left[45{}^{\circ}/0{}^{\circ}/-45{}^{\circ}/90{}^{\circ}\right]}_{\;2s}$ are produced. CPT is 125 μm for the M21 and 250 μm for the 977 based laminates. For both configurations a detailed investigation on the initiation and propagation of kink-band failure under compression is carried out. In order to have a defined damage state and to know the position of failure initiation, OHC tests are interrupted.

Laminate plates of 300 mm×300 mm are laid-up by hand from UD prepreg and cured in an autoclave at a maximum temperature of 180 °C and a maximum pressure of 7 bar with a curing cycle recommended for this prepreg by the manufacturer. Quality inspection of all laminates with ultrasound (US) inspection confirms that there are no manufacturing induced defects such as voids or delaminations. Specimens for UNC, OHC and CAI tests are cut from these plates with a diamond saw. For each test case, three or more plates of each configuration are produced in order to regard statistical variations of the manufacturing process within the test results.

The specimen width for UNC and OHC tests is according to aviation standard AITM 1-0008 [36] w=32 mm. Tab length is chosen to be 55 mm resulting in a total specimen length of l=142 mm. Aluminium and GFRP end tabs are applied on the specimen with 2-component epoxy adhesive (UHU Endfest-300), leaving the distance of the gauge length of l=32 mm between the tabs. The specimen side edges were polished to minimise edge effects. For the open-hole specimen, a hole with a diameter of d=6.34 mm is drilled through the thickness at the centre of test area.

The specimen geometry for CAI tests is according to ASTM-D-7136 [37] and ASTM-D-7137 [38] standard: length × width = 150 mm×100 mm. Impact damage is introduced in a drop weight tower by using a semi-spherical hardened steel striker with a diameter of 20 mm and a weight of 1.46 kg. Contact force is measured by a strain gauge full bridge. Anti-rebound after the first impact is ensured with a photo sensor activated clamp mechanism. All specimens are subjected with an impact of 10 J. Impact damage is assessed with US inspection and X-ray. US inspection is carried out by using a USPC 3040 DAC C-scan system (Dr. Hilger Ingenieurbüro, Aachen, Germany) with a resolution of 20 MHz and an amplification of up to 106 dB in 0.5 dB steps is used with a STS 10 MHz probe (Karl Deutsch GmbH, Wuppertal, Germany). For X-ray measurements a Faxitron Model 43855a (Faxitron Bioptics LLC, Tucson, AZ, USA) with the intensity set to 20 keV is used. Whereat Zinc iodide is applied as contrast medium to highlight cracks. In order to avoid moisture influence on the test results, all specimens are conditioned according to DIN EN 3615 [39].

### 2.2. Compression Test Set-Up

**Figure 2:**
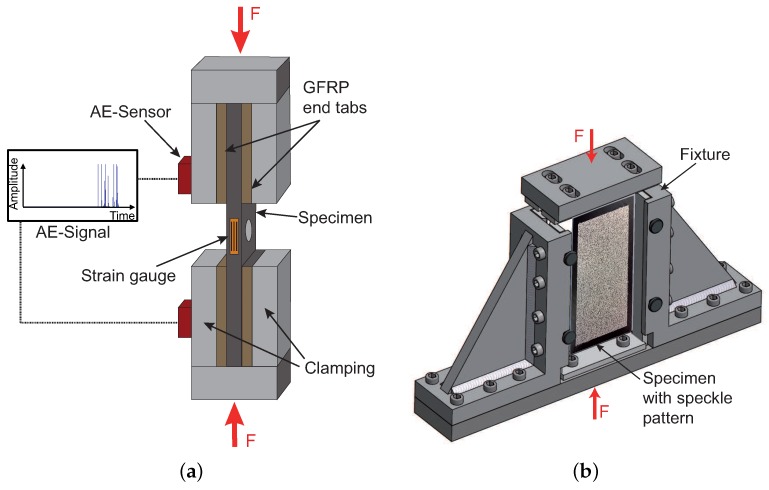
Scheme of the test set-up (**a**) for UNC and OHC tests, here shown for a specimen with a central hole and (**b**) for CAI tests.

Compression tests are executed by using a Zwick-Roell Z400 testing machine with a hydraulic pressure of 80bar damping the specimens at end tabs. Data is recording with the Zwick/Roell software *TestXpert*. The UNC and OHC specimens are mounted in a hydraulic composite compression fixture (HCCF) and are mainly loaded by compressive force on their end surfaces. Via the end tabs, a small amount of shear loading is applied as well. CAI tests are carried out according to the ASTM D-7137 [38]. Specimens are placed in a fixture as proposed in the standard. The cross-head speed is set to 0.5 mm/min for the UNC and OHC, respectively 1.0 mm/min for the CAI tests. The compressive load is measured by a 400 kN load cell, whereas displacement is measured via the traverse of the machine. The deformation and force are continuously recorded to determine elongation, compressive strength and the modulus of elasticity. Strain gauges are fixed on the specimen to measure local strain in the centre in order to determine whether global bending or out of plane buckling occurs. A scheme of both test set-ups is given in Figure 2, with the set-up for UNC, OHC tests in Figure 2a and for CAI tests in Figure 2b, respectively.

In order to determine failure initiation for interrupted test and for analysing differences regarding the damage process in the UNC and OHC tests, acoustic emission (AE) analysis is used. With AE analysis, acoustic waves generated at irreversible deformation of the material, i.e., cracking, are detected with piezoelectric sensors and the wave-signal is the conditioned and recorded by using by using amplifiers and filters and a respective software. One signal recorded this way is called a hit. The signal characteristics, such as the amplitude (the maximum peak in the AE signal waveform) can be analysed and correlated to damage mechanisms. The signal’s energy (the area under the squared signal envelope) is a measure for the accumulated elastic energy in the material released at deformation. The more severe the damage, the larger the energy released and recorded [40,41]. Detailed information about AE-analysis technique and principles can be found in the literature, i.e., [40,41]. The cumulative energy of the signals can be used to analyse instants at which larger cracks develop. Previous studies showed the usefulness of the AE-method for analysing failure mechanisms in composite laminates [42,43,44] or for determining the effects of voids on the compression behaviour in GFRP [45]. For the acoustic emission analysis, a Micro II multi-channel acquisition system from MISTRAS Group Inc. is used to record AE data. Two wideband differential (WD) sensors are used for AE wave detection. They are mounted on the surface of the HCCF clamping elements, using silicon grease as a coupling agent between the sensor and the fixture. This distant sensor attachment might result in a loss of AE-parameter accuracy, but is indispensable to protect the sensors from damage during the compression tests. Internal filters and a static threshold are used to reduce disturbance variables such as machine vibrations and ambient noise. The setting of the AE acquisition system is shown in Table 1. As the sound waves are disturbed and deflected at the interfaces on the way from the damage location to the sensor, frequency analysis or localisation results would be unreliable, but the test-set-up was successfully used before for comparing signal energy [45]. Before each test, a pencil lead break test with a Hsu-Nielsen Source [40,46] is carried out with the specimen mounted in the clamps in order to assure adequate mounting of the sensors. While testing, the AE-signal is used to stop the loading after the first peak in the amplitude signal for damage initiation analysis or after one of the following peaks for damage propagation analysis by using scanning electron microscopy (SEM) of polished micrograph sections.

The digital image correlation (DIC) system ARAMIS 4M (GOM, Braunschweig, Germany) with two cameras is used to analyse the strain field at the location of the impact in the CAI tests. For DIC measurements, a speckle-pattern is applied on one side of the specimen. In order to determine the damage initiation and propagation in detail, polished micrograph sections at characteristic damage states detected with AE-analysis are observed by SEM (Leo Gemini 1530, Zeiss, Oberkochen, Germany). The SE2 detector with a working distance between 5 mm and 7 mm at 2 keV is used. The edges of the samples are prepared with silver conductive paint to minimise charging issues, whereas renouncing the use of sputtering on the failure surface.

## 3. Results

### 3.1. Impact Damage

**Figure 3:**
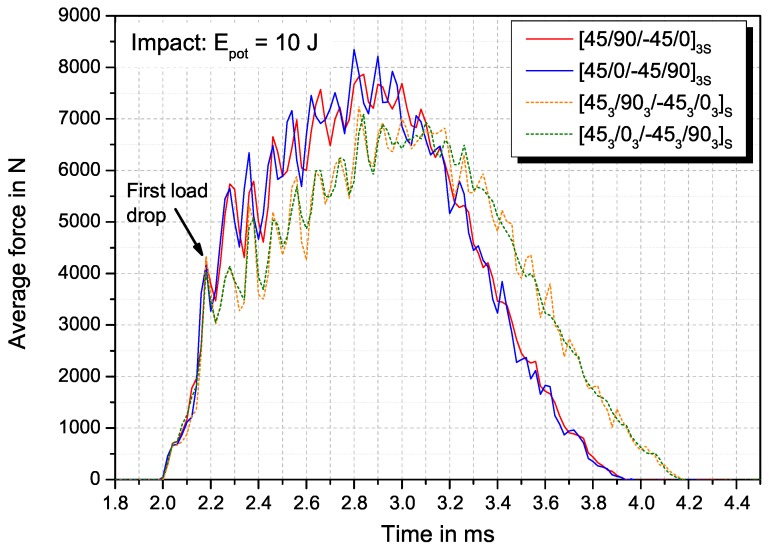
Average force-time curves during the impact event of the tested configurations (Material: HexPly-M21/34%/UD194/T800S).

Impact damage is evaluated by comparing the mechanical behaviour after impact, the projected damage area as well as delamination area in the interfaces between the layers. Figure 3 shows the average force-time curves for the four configurations at an impact event. The first load drop, referred to as the beginning of irreversible damage and deformation, is identical at approx. 4300 N force after 0.2 ms after the impactor hitting the surface. The further slope of the curve depends on the layer thickness. Thinner layers (sublaminate scaled) exhibit a steeper increase with a maximum impact force of 8300 N compared to 7800 N for the ply-block scaled laminate but a shorter contact time of 1.9 ms compared to 2.2 ms. The induced energy during the impact event is calculated induced energy calculated by integrating the respective force-time curve. The results of the impact tests are summarized in Table 2. In addition, the projected damage area measured from the backside echo of the US C-scans taken after the impact event is given. For the laminates with sublaminate-scaled stacking sequence, shorter contact time and higher maximum contact force are measured, indicating towards a higher stiffness in thickness direction and against bending during the impact event. Despite the slightly higher induced energy during the impact event, the projected damage area is significantly smaller (≈−65%) compared to ply-block scaled specimens.

### 3.2. Compression Tests

**Figure 4:**
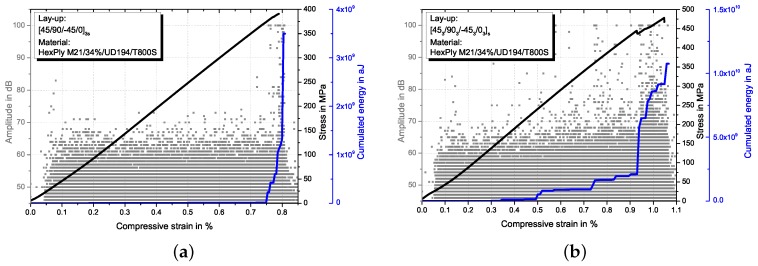
Representative OHC stress-strain curves and AE amplitude signals and cumulated energy over compressive strain for sublaminate (**a**) and ply-block (**b**) scaled specimens.

Linear stress-strain behaviour is observed until shortly before final failure occurs in UNC and OHC tests. Figure 4 shows representative OHC stress-strain curves together with AE amplitude signals and cumulated energy plotted over compressive strain for both, sublaminate (Figure 4a) and ply-block (Figure 4b) scaled specimens. For UNC, similar behaviour is observed. A slight decrease of the stress-strain curve slope is simultaneous to an increase in AE-signals of high amplitude, indicating the beginning of severe damage. For ply-block scaled laminates this damage initiation occurs at lower strains compared to the sublaminate scaled configuration. Sublaminate scaled specimens fail in a brittle way, whereas a more progressive failure with a higher amount of matrix cracks and delaminations before rupture is observed for ply-block scaled specimens. This is detectable with the AE amplitude signals. In sublaminate scaled specimens, AE-signals with amplitudes higher than 80 dB are detected only shortly before final failure, whereas for ply-block scaled specimens amplitudes higher than 80 dB are measured starting between 0.4% and 0.6% strain. The slope of the cumulated energy curves exhibit a sudden increase at the end of the test for sublaminate scaled and a continuous increase after approx. 0.5% strain for ply-block scaled specimens, confirming the more progressive failure of the latter. Results from UNC, OHC and CAI tests in terms of compressive strength and strain at initiation of severe damage $\varepsilon_{init}$ (detected with AE analysis) are summarised in Table 3. With DIC measurements, the buckling behaviour in the impact region is analysed. Ply-block scaled specimens exhibit larger buckling, especially for outer 0°-plies.

The strain at damage initiation depends on layer thickness. Thicker layers showing damage onset at lower strains and the observed progressive failure behaviour. In the UNC tests, a clear influence of layer thickness on compressive strength is observable. Thicker layers result on lower strength. In the UNC tests, the ply-thickness effect is more dominant than the influence of the stacking sequence. The position of the 0°-layers has no significant influence for ply-block case, where standard deviations overlap for specimens with inner or outer 0°-layers. For sublaminate scaling, higher UNC strength values are measured for specimens with inner 0°-layers.

In OHC tests, the position of the 0°-layers has high influence on the mechanical properties. Specimens with the 0°-layers inside exhibit significantly higher OHC strength with the highest value for the ply-block scaled configuration where all 0°-plies are concentrated in one thick layer in the centre of the specimen around the neutral plane (${[45{{}^{\circ}}_{3}/90{{}^{\circ}}_{3}/-45{{}^{\circ}}_{3}/0{{}^{\circ}}_{3}]\;}_{s}$). Note that this configuration would results in lower bending stiffness and strength compared to the other configurations investigated. However, in the OHC case the surrounding layers support the load bearing 0°-layers the most resulting in the highest measured OHC strength values.

**Figure 5:**
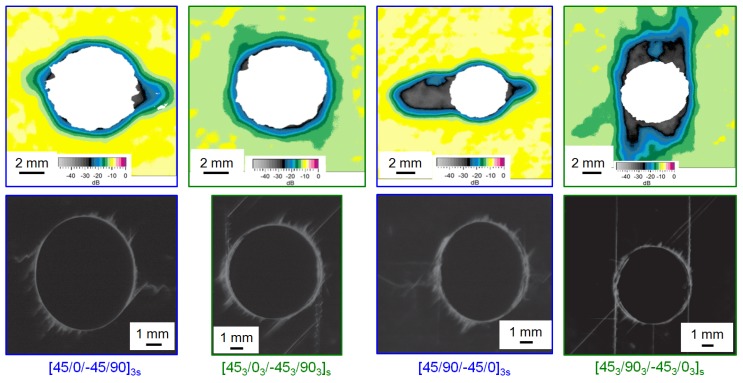
C-scan back-wall echo and X-ray images of failure initiation at approx. 0.7 % strain for representative OHC specimens.

In Figure 5 the damage initiation at the hole is compared by C-scan back-wall echo and X-ray images taken in interrupted tests. The specimens are tested until shortly before final failure occurs with the test being stopped at the first significant increase in high-amplitude AE-signals at to approx. 0.7% strain. The layer thickness determines the orientation of the first inter fibre failure (IFF) at the hole. IFF in sublaminate scaled specimens (thinner layers) occurs first in the 90°-layer transverse to loading direction whereas in the ply-block scaled specimens, IFF occurs first in the 90°-layers in loading direction.

**Figure 6:**
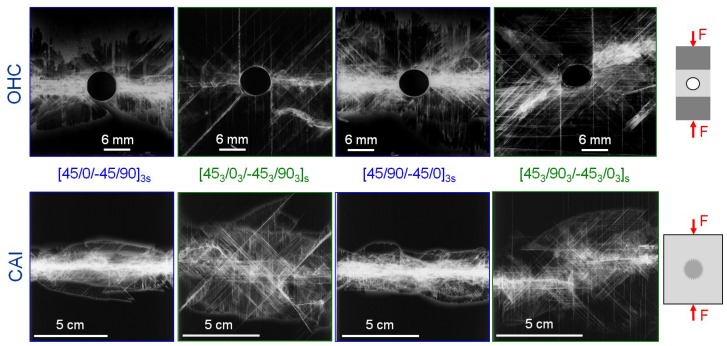
X-ray images of final failure for representative OHC (**top** row) and CAI (**bottom** row) specimens (Material: HexPly-M21/34%/UD194/T800S).

Regarding final failure, UNC specimens mostly fail in the gauge area between the tabs by a splitting type of damage accompanied by delaminations. Ply-block scaled specimens show larger delaminations at final failure, which corresponds to the progressive failure process with increased delamination growth before final failure, as it was observed and detected with AE-analysis. A kink-band can be visually identified at the line of splitting. All OHC specimens failed in the centre of the gauge area with a visible kink-band originating from the hole. Final failure of all CAI specimens originates at the impact damage. In both tests, ply block scaled specimens exhibit larger delamination damage than the sublaminate scaled at failure. The OHC failure process is analysed more in detail in Section 3.2 by using the results from the interrupted tests. X-ray images taken after final failure for representative OHC and CAI specimens of the four configurations are presented in Figure 6. Final failure initiates at the stress intensification, i.e., the hole or the impact damage, and is perpendicular to loading direction. For the sublaminate scaled specimens final failure occurs as brittle fracture in the 90° direction in a broad, split crack through the stress intensification that spans the width of the specimen. The stacking sequence or the shape of the stress intensification, cut out hole with clear sharp edges versus blunt impact damage, has no visible influence on final compressive failure. For the thick layers, the perpendicular crack broadens before leading to final failure, as can be seen in the images for the ply-block scaled specimens. Fracture is not strictly perpendicular to loading direction. Delamination and cracking fracture propagates at an angle of 45° from the stress intensification and then changes its orientation to 90° with regard to loading direction. In addition, larger delaminations are visible compared to the specimens with thinner layers.

**Figure 7:**
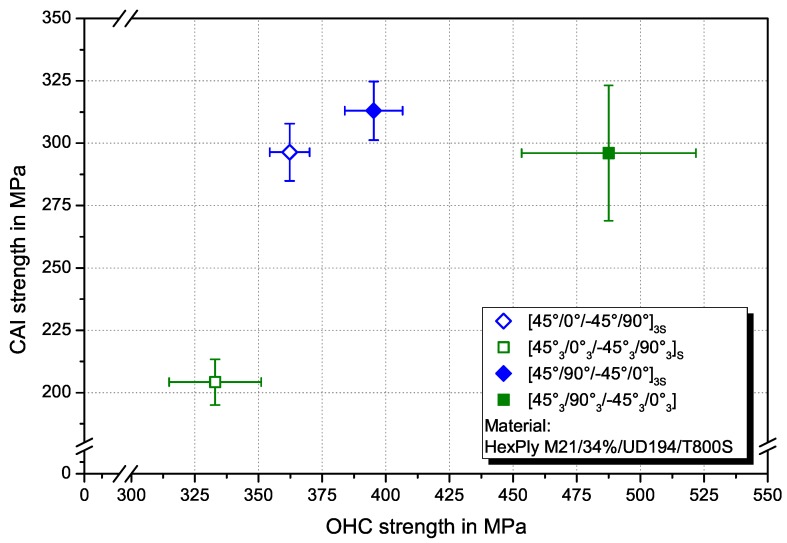
Comparison of CAI strength and OHC strength for the investigated configurations.

In Figure 7 the OHC and CAI strength with regard to the influence of ply thickness and stacking sequence (0°-layer position) are compared. Inner 0°-layers result in higher OHC and CAI strength for constant ply thickness. Although the configuration with six blocked layers in the central plane may not be favourable under bending or in the UNC case, it exhibits the highest CAI strength and only slightly lower OHC strength compared to the similar arranged sublaminate scaled configuration.

#### Interrupted Open-Hole Compression Tests

**Figure 8:**
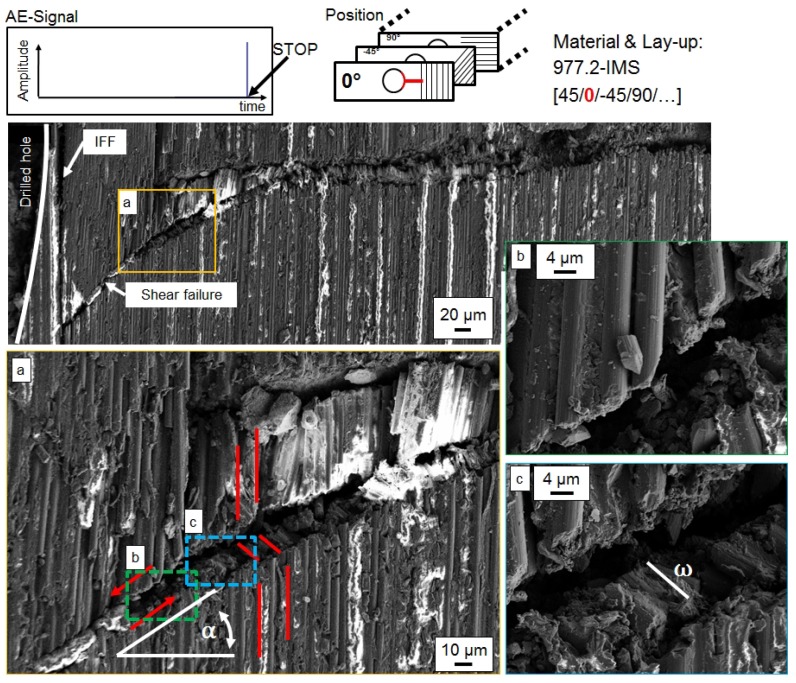
Damage initiation in the outer 0°-layer at the hole in a representative 977-2-IMS specimen.

**Figure 9:**
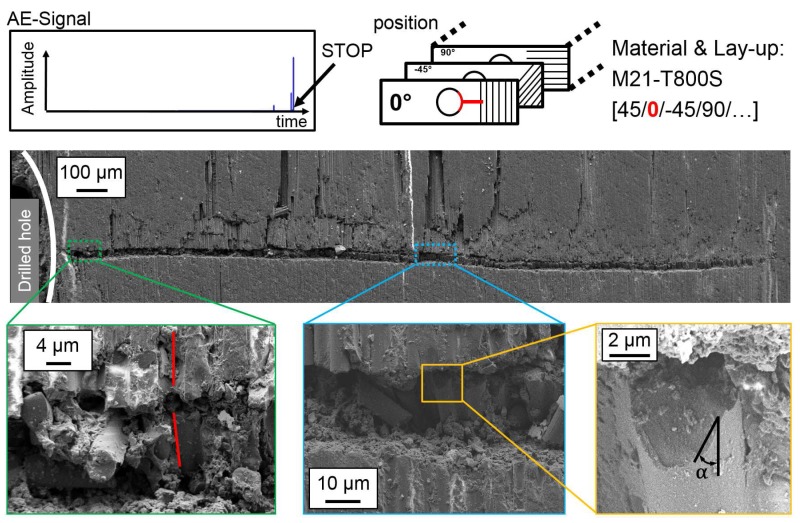
Damage initiation in the outer 0°-layer at the hole in a representative M21-T800S specimen.

In interrupted test compression tests, damage initiation and propagation at the hole is analysed with SEM images of polished micrograph sections. The position of the cut as well as the layer in the QI lay-up are highlighted in red within the figures. An overview of damage initiation at the hole and details of interesting sections in representative specimens are presented in Figure 8 for 977-2-IMS material (CPT = 250 μm) and in Figure 9 for M21-T800S material (CPT = 125 μm). For observation of damage initiation, the AE-signal is used to stop the loading after the first high peak in the amplitude signal, as indicated in the figures.

In 977-2-IMS specimen, a crack resulting from shear failure runs from the edge of the hole through the width of the 0°-layer. The crack originates at IFF parallel to the 0°-fibres and buckling of fibres into the hole is observed. The shear failure initiates a kink-band failure type at approx. 120 μm distance from the hole. Detail in Figure 8a shows very localised incipient kinking and the transition from shearing to kink-band failure (transient kinking). The shearing angle α that lies within 40° and the developed kink-band are marked in the image. The inclination angle Φ of fibres deflects in plane. The orientation angle β of the kink-band changes with damage progression. The shear failure and the kink-band with its angle $\omega \approx 2\times d_{f}$, where $d_{f}$ is fibre diameter, are shown at a higher magnification in Figure 8b, respectively Figure 8c. In the M21-T800S specimens, failure in the 0°-layer initiates at the edge of the hole as a straight crack perpendicular to loading direction (refer to Figure 9). The kink-band orientation angle β is constant (β=0) ant the inclination angle Φ of fibres deflects out of plane. No significant kink-band develops at this stage of damage, as can be seen in the details of higher magnification. The bottom right detail shows shear failure of a single fibre with the shearing angle α.

**Figure 10:**
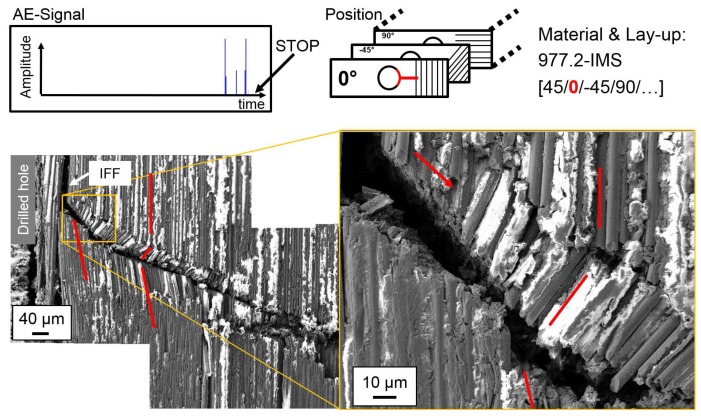
Damage propagation in the outer 0°-layer at the hole in a representative 977-2-IMS specimen.

**Figure 11:**
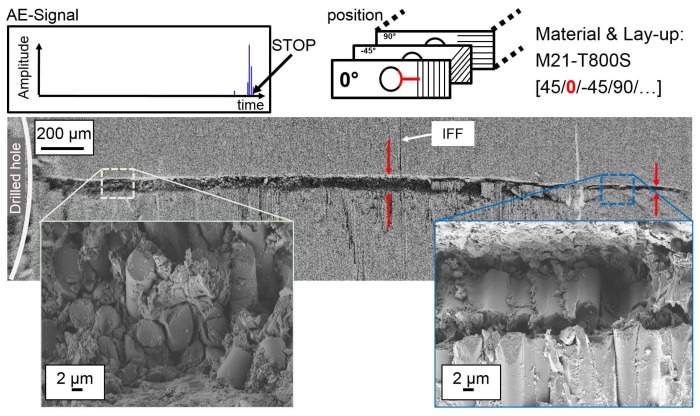
Damage propagation in the outer 0°-layer at the hole in a representative M21-T800S specimen.

**Figure 12:**
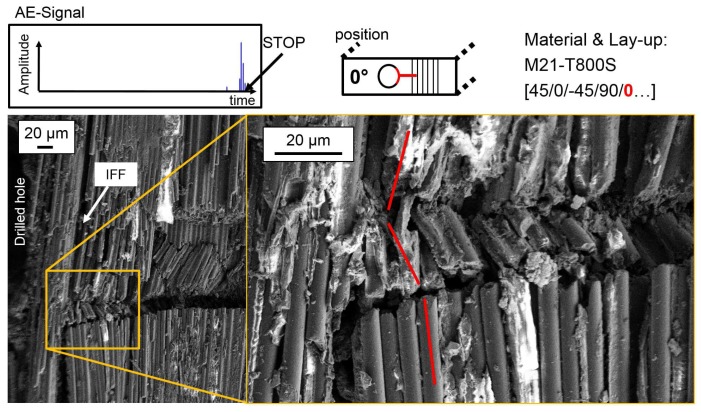
Damage propagation in the inner 0°-layer at the hole in a representative M21-T800S specimen.

For a damage propagation stage, the test is interrupted after one of the peaks in the AE amplitude signal following the first peak that indicates damage initiation. The AE-amplitude signals at the test stop are given in the figures. SEM images of polished micrograph sections of the outer 0°-layer are given in Figure 10 for 977-2-IMS material. For M21-T800S material, the influence of 0°-layer position is investigated as well. Figure 11 shows polished micrograph settings of the outer and Figure 12 of the inner 0°-layer during damage propagation.

Damage propagation in the outer 0°-layer of the 977-2-IMS material is visible as steady state kinking with kink-band broadening. IFF and buckling of fibres into the drilled hole is also observed. The inclination angle Φ of fibres is in plane and the kink-band angle β changes along the crack. In the outer 0°-layer of the M21-T800S specimens, no kink-band is visible, but the crack broadens and the amount of IFF between the 0°-fibres increases with increasing strain. Thus, two different width ω of the kink-band can be identified. Different fibre failure modes for single fibres, such as shear or tension failure are observed. In the inner 0°-layer, formation of a kink-band at local fibre misalignment due to IFF next to the hole is visible. This area is shown at a larger magnification in the detail in Figure 12. The inclination angle Φ of the fibres depends thus on the position of 0°-ply.

## 4. Discussion

Compression failure in FRP is dominated by the matrix properties. For the UNC, OHC and CAI tests, the same matrix system is used, to exclude this influence. The different matrix systems in the interrupted tests are very similar, so that the influence of the matrix properties for these tests is assumed to be small. Regarding the resistance against a low velocity impact, the smaller projected damage area of the sublaminate scaled laminates can be explained by the higher number of interfaces available for delamination. Induced energy at the impact is dissipated by matrix cracking and delamination damage. For these laminates, the delamination damage is distributed over 22 interfaces in the pine-tree shape, typical for impact damage in FRP [33]. For the ply-block scaled laminates only six interfaces between layers of different fibre orientation are available for delamination. As the induced energy is in the same range, it is dissipated by larger delaminations in contrast to a higher number of smaller size delaminations with increasing layer thickness. Due to the measuring principle, only the projected damage area and not the sum over all delaminated areas can be measured in the US c-scan analysis. This results in the significantly higher projected delamination area for the ply-block scaled specimens. The total summation of delamination area over all interfaces is assumed to be of equal size for both configurations. Similar findings are reported for thin-ply laminates compared to laminates with conventional ply thickness [21,30]. The size of the respective delamination areas and the fibre orientation next to the largest delamination is critical in the compression after impact test. A larger delamination area results in a weakening of the laminate against compression in general and a smaller support of the 0°-layers in particular due to the delamination crack opening being mode I dominated in compression. This explains the larger buckling, measured with the DIC, for ply-block scaled laminates with outer 0°-plies. The influence of the 0°-layer position, with inner 0°-layer exhibiting higher CAI strength, becomes clear, when considering the pine-tree delamination shape. Outer 0°-layers at the back sublaminate from the impact position are not supported over a comparable larger delamination area than inner 0°-layers. The unsupported length of the sublaminate increases due to delamination growth [33]. Consequently, the specimens have a lower resistance against global buckling, resulting in lower CAI strength with increasing delamination areas between the outer 0°-layers.

The damage onset at lower strains with increasing ply thickness observed in UNC and OHC tests is in accordance with results from literature for compressive [14] and tensile behaviour [20,21]. The higher UNC strength for sublaminate scaled specimens with inner 0°-layers can be attributed to a better support of the load bearing 0°-layers by adjacent plies, leading to a delay of the onset of microbuckling and the resulting kink-band initiation and rupture. This implies, that out-of-plane microbuckling is the dominating mechanisms for inner 0°-layers. This effect is more pronounced when a stress intensification is present. Here, the stacking sequence has a higher effect on strength than the layer thickness. Accordingly, the OHC and CAI strength is higher for inner 0°-layers layers. The highest CAI strength and comparably high OHC strength is measured for the configuration with one central 0°-layer consisting of six plies and surrounded by all other layers. Further investigations with thin-ply laminates might be necessary to verify these findings with thinner layers as a positive influence of a ply thickness below 50 μm is reported in literature [20,21,30]. However, regarding the results in Figure 7, decreasing the ply thickness down to thin-ply laminates ($t_{ply}\leq 40\;\mu m$) may not be the optimum in laminates with stress intensifications under pure compression loading. An open hole or impact damage reduces the bending resistance and has therefore a negative influence on global buckling. Central $0^{{}^{\circ}}$-layers increase the bending resistance under compression and show higher OHC strength. For CAI properties, central $0^{{}^{\circ}}$-layers are advantageous because of the conic delamination damage shape on an impact with the largest delaminations at the backside from the impact point. The delamination area at the load carrying $0^{{}^{\circ}}$-layers should be preferably small so that these layers should be arranged in the centre of a laminate. However, for bending load cases, outer 0°-layers are an optimum. Regarding the 0°-layer position, a trade-off between bending and compression strength has to be made.

OHC failure process depends on ply thickness, whereas the stacking sequence has no significant influence. Failure originates at the free edge of the hole in all tests and is perpendicular to loading direction in the 90°-direction for the ply-block scaled and in 0°-direction parallel to loading direction for the sublaminate scaled laminates. This can be explained by the difference in transverse contraction. Sublaminate scaled laminates with thinner layers fail brittle at comparable high strains whereas ply-block scaled laminates exhibit a damage process that initiates at lower strains and is more continuous and thus less brittle. Less brittle materials have a lower notch sensitivity, because early 0°-layer fibre matrix splitting in the ply-block scaled laminates may act as a blunting mechanism at the free edge [27]. Although a more progressive failure process might be advantageous in some materials, as it may result in the possibility to take measures for repair of replacement of the damaged part, a damage initiation at lower strains is mostly no acceptable for FRP as first ply failure is often a design criterion.

The polished micrograph sections from interrupted tests of representative specimens give insight on the influence of ply thickness on damage initiation and propagation at a free edge. The observed initiation of kink-band failure by shear driven damage mechanisms confirms the results from Gutkin et al. [7,8] for both, ply blocked and sublaminate scaled specimens. First damage of fibres occurs always in 0°-plies. Ply thickness and ply position have significant influence on the failure mechanisms. In ply-blocked specimens with thicker plies, a shear failure crack originates at IFF parallel to loading direction next to the free edge. The three stages of kinking [2,3] can be clearly identified in the SEM images. The matrix failure between the fibres supports fibre microbuckling and leads to shear driven failure and subsequently to localised incipient kinking of fibres (stage 1). The transient kinking region (stage 2) as the transition to a single dominant kink-band across the specimen is very small, leading to steady state kinking (stage 3) in close proximity to the hole. This kink-band is established almost instantly after damage initiation. With reduced ply thickness (sublaminate scaling) damage initiates and propagates as shear failure and a single kink-band is established at some distance away from the hole in a transition from the shear failure. This transition from incipient to transient and finally steady state kinking is shifted to higher compressive strains with decreasing ply thickness. Thinner plies exhibit a longer region of and a shear failure and more stable fracture process, which is less less prone to global buckling. This results in splitting final failure type observed in the UNC and OHC test. With increasing ply thickness, IFF in loading direction promotes formation of a single, stable kink-band and delaminations, leading to global buckling at lower strains. The observations regarding damage type and kink-band geometry are summarized in Table 4.

## 5. Conclusions

The experimental investigation on the influence of ply thickness and the position of the 0°-layers in QI CFRP laminates with a detailed analysis of the brittle failure process initiating at a stress concentration show the influence of the FRP lay-up on mechanical properties and damage propagation. This works thus may help to select an appropriate stacking sequence in the design of FRP parts with stress intensifications or where impact damage cannot be excluded regarding compression behaviour. When regarding free edges or an impact damage as delamination inducing stress intensifications within a laminate, the position of the 0°-layer is critical for stability under compression and is thus more important than the ply thickness. Central 0°-layers show best results for OHC and CAI strength due to higher bending stiffness and better supporting effect of the adjacent layers. Nonetheless, open-hole and CAI strength are higher for thinner layers, when regarding laminates with distributed 0°-plies. This is due to a reduced delamination area resulting an a shorter unsupported length of the load bearing sublaminates. The statistical defect distribution and the increased in situ strength lead to a delayed damage initiation with decreasing ply thickness. Thus, the unnotched compressive strength increases with decreasing ply thickness. With increasing ply thickness, damage initiation in the form of IFF is at lower strains. This reduces the stress concentration factor at the stress intensification and leads to a change from brittle to progressive delamination failure. In laminates with blocked plies, final failure is transverse to loading direction with an orientation along the $\pm 45^{{}^{\circ}}$-layers around the stress intensification that leads to a step in the fracture plane, whereas in laminates with distributes plies, final fracture occurs as one straight splitted crack transverse to loading direction.

Damage initiation and propagation at stress intensifications, such as the free edges in open-hole specimens and the impact damage in the case of CAI, is analysed with AE analysis, X-ray and polished micrograph sections in SEM of representative specimens. AE signals are used in interrupted OHC tests for causing a distinct damage state and examine it at a precise instant of time during fracture process. Furthermore, characteristic plots for different types of damage propagation highlight the potential of the AE-analysis for monitoring failure in composites.

First damage of fibres occurs always in a 0°-ply. Fibre shear failure leads to local microbuckling and the formation and growth of a kink-band as final failure mechanisms. Ply thickness and position of ply influence the failure mechanism. The formation of a kink-band and finally steady state kinking is shifted to higher compressive strains with decreasing ply thickness. Final failure mode in laminates with stress intensification depends on ply thickness. In thick or inner plies, damage initiates as shear failure with an in-plane inclination angle ω and fibre buckling into the drilled hole (free edge). The kink-band orientation angle β is changing with increasing strain. In outer or thin plies we observed shear failure of single fibres as first damage and an out-of-plane inclination angle ω. The kink-band orientation angle β is constant until final failure. Further investigations should focus on thin-ply laminates that offer improved mechanical properties due to a dramatic reduction in ply thickness and allow a broader variety of lay-up configurations.

## Figures and Tables

**Table 1:** Parameters of the AE acquisition system (Sensor: WD).

Parameter	Value
Frame rate/MHz	5
Preamp gain/dB	40
Threshold/dB	45
Hit definition time (HDT)/μs	200
Hit lockout time (HLT)/μs	300
Peak definition time (PDT)/μs	50
Maximum hit duration/μs	100

**Table 2:** Results of impact test and boundary conditions.

	${\left[45/0/-45/90\right]}_{3s}$	${\left[45_{3}/0_{3}/-45_{3}/90_{3}\right]}_{s}$	${\left[45/90/-45/0\right]}_{3s}$	${\left[45_{3}/90_{3}/-45_{3}/0_{3}\right]}_{s}$
Contact time/ms	1.90±0.02	2.14±0.05	1.92±0.02	2.16±0.03
Max. contact force/N	8552.30±104.00	7292.50±111.60	8217.10±240.30	7484.00±322.90
Induced energy/J	5.48±0.20	4.92±0.64	5.21±0.32	5.15±0.35
Damage area/${\mathrm{mm}}^{2}$	650.00±79.00	1894.00±129.00	646.00±26.00	1937.00±46.00

**Table 3:** Test results from UNC, OHC and CAI tests. Strain at initiation of severe damage $\varepsilon_{init}$ is defined as the strain value at which first AE-signals with amplitudes >80 dB occur.

	${\left[45/0/-45/90\right]}_{3s}$	${\left[45_{3}/0_{3}/-45_{3}/90_{3}\right]}_{s}$	${\left[45/90/-45/0\right]}_{3s}$	${\left[45_{3}/90_{3}/-45_{3}/0_{3}\right]}_{s}$
UNC strength/MPa	681.47±26.80	533.26±67.95	639.72±23.49	563.74±34.82
UNC $\varepsilon_{init}$/%	≈1.40	≈0.70	≈1.60	≈0.80
OHC strength/MPa	362.24±7.33	336.36±16.51	398.94±10.38	487.57±31.22
OHC $\varepsilon_{init}$/%	≈0.70	≈0.60	≈0.77	≈0.50
CAI strength/MPa	296.36±11.46	204.23±9.14	313.00±11.76	296.04±27.09

**Table 4:** Observations on damage type and kink-band geometry in dependency of ply thickness and ply position within the laminate.

Thick ply	Inner ply	Thin ply	Outer ply
Shear failure		Shear failure of single fibre	
Φ in plane		Φ out of plane	
β is changing		β is constant	

## Abbreviations

The following abbreviations are used in this manuscript:

AE	Acoustic emission
CAI	Compression after impact
CFRP	Carbon fibre reinforced plastic
DIC	Digital image correlation
CPT	Cured ply thickness
OHC	Open-hole compression
QI	Quasi-isotropic
SEM	Scanning electron microscopy
UNC	Unnotched Compression
US	Ultrasonic

## References

[1] Rosen B.W. (1964). Mechanics of composite strengthening. American Society for Metals: Seminar on Fiber Composite Materials.

[2] Moran P.M., Liu X.H., Shih C.F. (1995). Kink band formation and band broadening in fiber composites under compressive loading. Acta Metall. Mater..

[3] Poulsen J.S., Moran P.M., Shih C.F., Byskov E. (1997). Kink band initiation and band broadening in clear wood under compressive loading. Mech. Mater..

[4] Fleck N., Jelf P., Curtis P. (1995). Compressive Failure of Laminated and Woven Composites: Compressive Failure of Laminated and Woven Composites. J. Compos. Technol. Res..

[5] Budiansky B., Fleck N.A. (1993). Compressive failure of fibre composites. J. Mech. Phys. Solids.

[6] Budiansky B., Fleck N.A., Amazigo J.C. (1998). On kink-band propagation in fiber composites. J. Mech. Phys. Solids.

[7] Gutkin R., Pinho S.T., Robinson P., Curtis P. (2010). On the transition from shear-driven fibre compressive failure to fibre kinking in notched CFRP laminates under longitudinal compression. Compos. Sci. Technol..

[8] Gutkin R., Pinho S.T., Robinson P., Curtis P.T. (2010). Micro-mechanical modelling of shear-driven fibre compressive failure and of fibre kinking for failure envelope generation in CFRP laminates. Compos. Sci. Technol..

[9] Liebig W.V., Leopold C., Schulte K. (2013). Photoelastic study of stresses in the vicinity of a unique void in a fibre-reinforced model composite under compression. Compos. Sci. Technol..

[10] Liebig W.V., Schulte K., Fiedler B. (2016). Hierarchical analysis of the degradation of fibre-reinforced polymers under the presence of void imperfections. Philos. Trans. Ser. A Math. Phys. Eng. Sci..

[11] Shams S.S., Elhajjar R.F. (2015). Investigation into the effects of fiber waviness in standard notched composite specimens. CEAS Aeronaut. J..

[12] Wisnom M.R. (1999). Size effects in the testing of fibre-composite materials. Compos. Sci. Technol..

[13] Soutis C. (1991). Measurement of the static compressive strength of carbon-fibre/epoxy laminates. Compos. Sci. Technol..

[14] Lee J., Soutis C. (2005). Thickness effect on the compressive strength of T800/924C carbon fibre—Epoxy laminates. Compos. Part A Appl. Sci. Manuf..

[15] Lee J., Soutis C. (2008). Measuring the notched compressive strength of composite laminates: Specimen size effects. Compos. Sci. Technol..

[16] Soutis C., Lee J. (2008). Scaling effects in notched carbon fibre/epoxy composites loaded in compression. J. Mater. Sci..

[17] Wisnom M.R., Hallett S.R., Soutis C. (2010). Scaling Effects in Notched Composites. J. Compos. Mater..

[18] Arteiro A., Catalanotti G., Melro A.R., Linde P., Camanho P.P. (2015). Micro-mechanical analysis of the effect of ply thickness on the transverse compressive strength of polymer composites. Compos. Part A Appl. Sci. Manuf..

[19] Flaggs D.L., Kural M.H. (1982). Experimental Determination of the In Situ Transverse Lamina Strength in Graphite/Epoxy Laminates. J. Compos. Mater..

[20] Sihn S., Kim R.Y., Kawabe K., Tsai S.W. (2007). Experimental studies of thin-ply laminated composites. Compos. Sci. Technol..

[21] Amacher R., Cugnoni J., Botsis J., Sorensen L., Smith W., Dransfeld C. (2014). Thin ply composites: Experimental characterization and modeling of size-effects. Compos. Sci. Technol..

[22] Pipes R.B., Pagano N.J. (1970). Interlaminar Stresses in Composite Laminates Under Uniform Axial Extension. J. Compos. Mater..

[23] Pagano N.J., Pipes R.B. (1971). The Influence of Stacking Sequence on Laminate Strength. J. Compos. Mater..

[24] Mittelstedt C., Becker W. (2007). Free-Edge Effects in Composite Laminates. Appl. Mech. Rev..

[25] Camanho P.P., Arteiro A., Turon A., Costa J., Guillamet E.G. (2012). Structural integrity of thin-ply laminates. JEC Compos. Mag..

[26] Soutis C., Fleck N.A., Smith P.A. (1991). Failure Prediction Technique for Compression Loaded Carbon Fibre-Epoxy Laminate with Open Holes. J. Compos. Mater..

[27] Erçin G.H., Camanho P.P., Xavier J., Catalanotti G., Mahdi S., Linde P. (2013). Size effects on the tensile and compressive failure of notched composite laminates. Compos. Struct..

[28] Suemasu H., Takahashi H., Ishikawa T. (2006). On failure mechanisms of composite laminates with an open hole subjected to compressive load. Compos. Sci. Technol..

[29] Wang J., Callus P., Bannister M. (2004). Experimental and numerical investigation of the tension and compression strength of un-notched and notched quasi-isotropic laminates. Compos. Struct..

[30] Saito H., Morita M., Kawabe K., Kanesaki M., Takeuchi H., Tanaka M., Kimpara I. (2011). Effect of ply-thickness on impact damage morphology in CFRP laminates. J. Reinf. Plast. Compos..

[31] González E.V., Maimí P., Camanho P.P., Lopes C.S., Blanco N. (2011). Effects of ply clustering in laminated composite plates under low-velocity impact loading. Compos. Sci. Technol..

[32] González E.V., Maimí P., Camanho P.P., Turon A., Mayugo J.A. (2012). Simulation of drop-weight impact and compression after impact tests on composite laminates. Compos. Struct..

[33] Bull D.J., Spearing S.M., Sinclair I. (2014). Observations of damage development from compression-after-impact experiments using ex situ micro-focus computed tomography. Compos. Sci. Technol..

[34] Yokozeki T., Aoki Y., Ogasawara T. (2008). Experimental characterization of strength and damage resistance properties of thin-ply carbon fiber/toughened epoxy laminates. Compos. Struct..

[35] Greenhalgh E.S., Rogers C., Robinson P. (2009). Fractographic observations on delamination growth and the subsequent migration through the laminate. Compos. Sci. Technol..

[36] Airbus S.A.S. (2004). AITM 1-0008: Fibre Reinforced Plastics—Determination of Plain, Open Hole and Filled Hole Compression Strength.

[37] ASTM International (2015). ASTM D7136 / D7136M-15: Test Method for Measuring the Damage Resistance of a Fiber-Reinforced Polymer Matrix Composite to a Drop-Weight Impact Event.

[38] ASTM International (2012). ASTM D7137 / D7137M-12: Test Method for Compressive Residual Strength Properties of Damaged Polymer Matrix Composite Plates.

[39] German Institute for Standardisation (1999). DIN EN 3615:1999-04: Fibre Reinforced Plastics—Determination of the Conditions of Exposure to Humid Atmosphere and of Moisture Absorption (Withdrawn).

[40] Große C.U., Ohtsu M. (2008). Acoustic Emission Testing: Basics for Research—Applications in Civil Engineering.

[41] Mizutani Y., Mizutani Y., The Japanese Society for Non-Destructive Inspection (2016). Practical Acoustic Emission Testing.

[42] Qing-Qing N., Eiichi J. (1993). Acoustic emission and fracture of Carbon Fiber Reinforced Thermosoftening Plastic (CFRTP) materials under monotonous tensile loading. Eng. Fracture Mech..

[43] Mizutani Y., Nagashima K., Takemoto M., Ono K. (2000). Fracture mechanism characterization of cross-ply carbon-fiber composites using acoustic emission analysis. NDT E Int..

[44] Liu P.F., Chu J.K., Liu Y.L., Zheng J.Y. (2012). A study on the failure mechanisms of carbon fiber/epoxy composite laminates using acoustic emission. Mater. Des..

[45] Kosmann N., Karsten J., Schuett M., Schulte K., Fiedler B. (2015). Determining the effect of voids in GFRP on the damage behaviour under compression loading using acoustic emission. Compos. Part B Eng..

[46] Hsu N., Breckenridge R. (1981). Characterization and Calibration of Acoustic Emission Sensors. Mater. Eval..

